# Influence of Estrogens on Uterine Vascular Adaptation in Normal and Preeclamptic Pregnancies

**DOI:** 10.3390/ijms21072592

**Published:** 2020-04-08

**Authors:** Maurizio Mandalà

**Affiliations:** Department of Biology, Ecology and Earth Sciences, University of Calabria, 87036 Rende, Italy; m.mandala@unical.it

**Keywords:** pregnancy, preeclampsia, estrogens, estrogen-receptors, uterine vascular vasodilation

## Abstract

During pregnancy, the maternal cardiovascular system undergoes significant changes, including increased heart rate, cardiac output, plasma volume, and uteroplacental blood flow (UPBF) that are required for a successful pregnancy outcome. The increased UPBF is secondary to profound circumferential growth that extends from the downstream small spiral arteries to the upstream conduit main uterine artery. Although some of the mechanisms underlying uterine vascular remodeling are, in part, known, the factors that drive the remodeling are less clear. That higher circulating levels of estrogens are positively correlated with gestational uterine vascular remodeling suggests their involvement in this process. Estrogens binding to the estrogen receptors expressed in cytotrophoblast cells and in the uterine artery wall stimulate an outward hypertrophic remodeling of uterine vasculature. In preeclampsia, generally lower concentrations of estrogens limit the proper uterine remodeling, thereby reducing UPBF increases and restricting the growth of the fetus. This review aims to report estrogenic regulation of the maternal uterine circulatory adaptation in physiological and pathological pregnancy that favors vasodilation, and to consider the underlying molecular mechanisms by which estrogens regulate uteroplacental hemodynamics.

## 1. Introduction

Progressive and significant blood flow increases to the uterus (and placenta) are required for suppling adequate oxygen and nutrients for fetoplacental growth and development, and for normal pregnancy outcomes. In different species, such as the *ewe, rabbit, rat, mouse, guinea pig*, and *humans*, measurements of uteroplacental blood flow (UPBF) consistently show many-fold increases occurring during pregnancy. In women, UPBF increases from 20–50 mL/min in the nonpregnant state up to 450–800 mL/min by late gestation [1]. Insufficient UPBF to the gravid uterus is associated with pregnancy complications such as preterm delivery, intrauterine growth restriction (IUGR) and preeclampsia (PE) [2,3]. The latter is a worldwide disease that affects 3–8% of pregnant women, and is characterized by hypertension, proteinuria, and edema, which may cause fetal growth restrictions and health complications in later life for both mother and infant [4]. The well-recognized clinical complications of PE underlie the pivotal importance of a proper rise of UPBF during pregnancy.

In normal pregnancy, the increase in UPBF is facilitated by significant changes in both the structure and function of the entire maternal uterine vasculature [5]. The growth in arterial and venous axial length and circumference lowers uterine vascular resistance and increases capacitance. Further, in pregnant women, total maternal blood volume and cardiac output rise about 40% by term [6,7] and the uterine vessels carry up to 25% of the cardiac output vs. less than 1% in the nonpregnant state [6,8]. This profound maternal physiological adaptation to pregnancy is required for a successful pregnancy in terms of both fetal and maternal wellbeing.

The uterine vascular changes associated with pregnancy begin in early gestation, before placentation is completed, with the cytotrophoblast cell invasion of maternal spiral arteries [9,10], which is considered to be the mechanical trigger for initiating the uterine vascular transformation. The crucial role of spiral artery invasion for the uterine vascular changes is evidenced by the fact that a shallow invasion does not allow adequate transformation and a sufficient UPBF, as observed in PE, which is characterized by higher peripheral vascular resistance compared with normal pregnancy.

Investigations for several decades have been carried out to determine gestational changes in the structure and function of the uterine vasculature, and to understand the underlying mechanisms. The uterine vascular changes occur at different levels: (a) endothelial cells (EC), the lining cell of the vessels, (b) smooth muscle cells (SMC) in the medial layer, and c) the extracellular matrix. Although the extent of uterine vascular growth during pregnancy is well-studied, the factors that induce and regulate this process are still not completely known. Data from the literature suggests several actors take part in the complex process underlying uterine vascular remodeling.

The fact that plasma concentrations of estrogens increase significantly during gestation has prompted investigators to explore how this newly-instituted endocrine environment associated with pregnancy may assist in the remodeling of maternal uterine vasculature. In the current article, the author reviews the estrogenic mechanisms underlying uterine vascular vasodilation and remodeling, and the evidence that estrogens participate in this process by regulating multiple mechanisms and interactive pathways.

## 2. Uterine Circulation during Pregnancy

Uterine hemodynamic changes during pregnancy result in a significant augmentation of UPBF, necessary for fetal sustenance and growth, a process that is accomplished by a combination of substantially increased maternal blood volume and cardiac output and decreased uterine vascular resistance. Direct measurement of UPBF in animals, as well as in women with Doppler Ultrasound, show a great increase from the nonpregnant vs. pregnant state, with species-dependent changes in pattern and magnitude. The increase is gradual and fairly linear in women [11], as well as in *guinea pigs* [12], with an increase of >10-fold. In other species, such as *rodents* [13], *primates* [14] and *sheep* [15], the increase can be even higher, up to 100-fold, and with a less linear pattern, e.g., 90% near term but less than 10% at mid pregnancy.

The clinical relevance of the increased UPBF during pregnancy is underscored by the fact that its aberration leads to common pregnancy pathologies such as preterm labor, intrauterine growth restriction (IUGR), and PE. In preeclamptic women, the magnitude of increase in UPBF is reduced with consequent IUGR often documented [16]. A significantly reduced UPBF has been reported in pregnant women at high-altitude and is associated to a poor fetal growth, which increases mortality and morbidity [17]. A study in pregnant *sheep* exposed to high-altitude suggested that, in this condition, the reduction in UPBF is due to vasodilation and hypoxic inhibition of estrogen-induced activation of extracellular signal-regulated Kinase 1/2 (ERK1/2) [18].

A long and productive history of *human* studies from Moore et al. has demonstrated that chronic hypoxia interferes with the maternal circulatory adjustments to pregnancy and results in restricted uterine arterial expansive growth and decreased UPBF. However, a different susceptibility to altitude-related damage has been demonstrated among populations in relation to their duration of altitude exposure, with highland natives demonstrating a unique pregnancy-hemodynamic response that contributes to their ability to protect against altitude-associated IUGR [19,20]. This suggests the involvement of both genetic and environmental factors in estrogen-uterine vascular remodeling during pregnancy.

That estrogens may play a role in the regulation of UPBF is suggested by the positive correlation between its plasma levels and UPBF during physiologic states that have high uterine blood flow, such as pregnancy and the follicular phase of the ovarian cycle [21,22]. In addition, exogenous estrogen has a similar effect, for example, administration of estradiol-17 beta to nonpregnant ovariectomized *ewes* induces uterine vasodilation and increases UPBF considerably, an effect that was reduced by specific inhibition of estrogen receptors (ERs) [23,24]. The increased UPBF during pregnancy is secondary to dramatic vasodilation of uterine vasculature together with other uterine vascular events associated with pregnancy such as vasculogenesis and angiogenesis. [25,26,27].

Estrogens may drive uterine vascular remodeling in multiple ways, e.g., by (1) favoring development and cytotrophoblast cell invasion of spiral arteries [9,10]; (2) activating endothelial production of relaxation factors such as nitric oxide (NO), [28] and prostacyclin (PGI2) [29]; (3) modifying ion channel activity and expression in EC [30] and SMC [31]; (4) inducing changes in the vascular matrix [32]; and (5) stimulating the proliferation and migration of uterine EC by upregulation of pro-angiogenic factors such as vascular endothelial growth factor (VEGF) and placental growth factor (PlFG), and their receptors VEGFR1 and VEGFR2 [33,34,35,36]. Estrogens regulate all these processes by activating multiple pathways, e.g., those that involve phosphatidylinositol-3 kinase/protein kinase, *c-Fos*, *c-Jun*, and *Sp1* [37,38]. Due to limited space, these will not be discussed further in this review.

## 3. Maternal Gestational Uterine Vascular Remodeling

Considerable plasticity of the maternal cardiovascular system occurs during pregnancy. It includes physiological and anatomical changes that result in increased cardiac output, blood volume, and vascular compliance and in reduced blood pressure secondary to a decreased peripheral vascular resistance [39,40,41]. As already mentioned, during pregnancy, the uterine circulation must undergo profound changes in structure and function to provide adequate blood to the growing uterus and fetoplacental unit. This is accomplished by both arteries and veins expanding in length and circumference (or diameter), and also by changes in the vascular wall at the cellular level (EC and SMC) and in matrix composition.

Several studies underscore the fact that uterine vascular remodeling begins with the cytotrophoblast invasion of smaller vessels just proximal to the site of placentation (spiral arteries) and, as pregnancy progresses, proceeds to the larger more upstream vessels [42,43]. In early pregnancy, cytotrophoblast cells proliferate and migrate into the decidua, the myometrium, and finally into maternal spiral arteries [44,45]. Endovascular cytotrophoblast invasion modifies the muscular wall of the spiral arteries, ablating endothelial and smooth muscle cells and transforming these vessels from high- to low-resistance channels [46]. This results in increasing blood flow to the intervillous space, an event which increases shear stress in upstream uterine vessels and raises the production of other endothelial-derived relaxation factors such as PGI2, VEGF, and PLGF. This series of events facilitates the profound uterine vasodilation, increased capacitance, and augmented UPBF.

Much of our understanding of the events that underlie uterine vascular remodeling comes from in vitro studies on isolated maternal uterine arteries and arterioles. Studies in different species including *humans* have documented growth of both large (conductive) and small (resistance) vessels with increases in diameter, length, and cross-sectional area, suggestive of outward hypertrophic remodeling [47,48,49,50]. In women, comparison of uterine vessels in the nonpregnant state vs. those in late pregnancy reveals an approximate doubling of the radius [51]. This would theoretically increase flow by about 16-fold as suggested by Poiseuille’s law that states that flow is proportional to the quadratic of the radius. Vascular distensibility also increases during pregnancy due to altered extracellular matrix composition, most likely due to changes in the activity of matrix metalloproteinases [32,48,52].

Uterine vascular remodeling during pregnancy is a complex, active process resulting from multiple cellular signals and mechanisms. The remodeling is due to the combined influence of local and systemic factors; in particular, the major humoral influences include sex steroids (estrogens and progesterone) secreted by the fetoplacental unit. Defining the remodeling process is essential for better understanding the physiologic regulation of maternal uterine blood flow and how it is impacted under pathophysiological conditions.

## 4. Estrogens in Pregnancy

In pregnant *mammals*, estrogens are produced mainly by the ovaries and, in smaller amounts, by adrenal glands and adipose tissue [53]. In women, placental production of estrogens surpasses that of the corpus luteum in the ovary around 9 weeks, an event termed the luteoplacental shift [54]. In particular, the syncytiotrophoblast cell layer differentiated from cytotrophoblast cells is the site of estrogen production during pregnancy [55]. The synthesis of estrogens is mainly derived from aromatization of dehydroepiandrosterone (DHEA), D4-androstenedione, and testosterone. DHEA is produced by maternal and fetal adrenal glands that transform cholesterol into pregnenolone and then DHEA by the enzymes cytochrome P450 steroid chain cleavage (CYP450scc) and 17α-hydroxylase,17,20 lyase (CYP17A1), respectively.

Also, some local synthesis of estrogens occurs in uterine artery smooth muscle cells (UASMC) as suggested by the presence of aromatase [56,57]. Estrogen synthesis during pregnancy includes estrone (E1), estradiol (E2) (most abundant and potent), and estriol (E3), with some interconversion between them occurring along with hydroxylation into estetrol (E4), catecholestrogens, methoxyestrogens, and conjugated forms, a family of active molecules with genomic and non-genomic effects [30,58,59,60,61]. Estrogens can also be metabolized into inactive hormones and excreted into the bile and/or urine to prevent complications from an excess of circulating estrogens [62,63].

The maternal plasma concentration of estrogen increases progressively during pregnancy from 367 pM (luteal phase) up to 11,000–37,000 pM at term [64,65]. Estrogens act in several tissues via specific ERs that help bring about the physiological changes associated with pregnancy.

Several studies have demonstrated a role for estrogens in promoting angiogenesis and vasodilation of the uterine vasculature with a consequent profound increase in UPBF [15,29]. Their effects are mediated by genomic and non-genomic signaling pathways, such that estrogens regulate gene expression by interacting directly with specific DNA sequences or acting indirectly through different transcription factors such as *Sp1* or the *c-Fos/c-Jun* complex, which is also called activated protein 1 [66,67,68]. Non-genomic effects are exerted through pathways involving key signaling molecules such as protein kinases, NOS, COX, and growth factors [28,69,70].

### Estrogen Receptors (ERs)

In the reproductive system, ERs are present in the placenta, ovaries, uterus, and uterine artery (UA) [70,71,72,73]. Several types of ERs have been identified, including ERα and ERβ (mainly nuclear receptors) and G protein-coupled estrogen receptor (GPER, a membrane receptor) and their isoforms; all of them bind estrogens with different affinities and different kinetics of dissociation [58,74].

Classical, ligand-activated genomic effects of ERs were once thought to mediate all estrogen responses, however, it is now accepted that rapid, non-genomic responses are mediated by ER-containing membrane complexes that occur in many tissues. The uterine vasculature contains ERs and responds to estrogen via both genomic and non-genomic mechanisms [24,75,76] in EC and in SMC of UA, and their expression is upregulated by pregnancy [77,78].

Estrogen binding to ERs induces angiogenesis and vasodilation to mediate the increases in UPBF that occur during the follicular phase and in pregnancy: Two physiological states characterized by high circulating estrogen concentrations [78]. Most studies focused on understanding the molecular mechanism of estrogen-relaxation have used *ovine* UA and have shown that estrogens act as potent vasodilators in the regulation of the uterine vascular tone. [79,80]. Besides the regulation of vascular tone, estrogens also affect UA structure as demonstrated in women UA where an overexpression of ERα was correlated with lower collagen concentrations, making the vessels more distensible [81].

## 5. Estrogen Influences on Maternal Uterine Vascular Remodeling

The role of the estrogens in the onset of uterine vascular remodeling during pregnancy has been supported by studies using mainly two different experimental approaches: induction of pseudopregnancy and hormone replacement in ovariectomized animals. Pseudopregnancy, a condition that can be induced in some animal species by sexual stimulation, increases circulating sex steroids similar to the levels seen in early- and mid-pregnancy, and is associated with increased lumen diameter, cross-sectional area, and proliferation of uterine medial SMC [82]. Also, ovariectomized animals injected with estradiol display uterine structural and cellular changes similar to those seen in estrus and even more similar to those seen in pregnancy [83,84]. In support of the concept that estrogens are involved in pregnancy-related changes in uterine vessels, at least in part, is that elevated endogenous estrogen levels in physiological states such as estrus and pregnancy, or via subcutaneously-administrated exogenous estrogen, induces DNA synthesis and UA growth [83].

Estrogens induce changes in the uterine vasculature during pregnancy directly or indirectly by modulating numerous cellular processes such as growth and remodeling, vascular contractility, and matrix deposition [85].

### 5.1. Estrogens Induce Cytotrophoblast Invasion of Spiral Arteries

As already noted, the initiation of uterine vascular remodeling is often ascribed to the endovascular cytotrophoblast invasion of the spiral arteries. Cytotrophoblast-orchestrated spiral artery remodeling is central to normal pregnancy and has been suggested by several investigators to be under the influence of estrogens [44,45]. In normal placentas, the high expression of estrogen receptors (ERα & ERβ) on cytotrophoblast cells make them a sensitive target for estrogens that stimulate differentiation in extra-villous trophoblast and migration into the spiral arteries. Although it is not clear how this process is regulated and limited, a study in pregnant *baboon* shown that increasing circulating estrogens concentrations between the second and third trimester can inhibit cytotrophoblast advancement, probably by upregulation of VEGF expression [86]. Also, immune cells, mainly uterine natural killer (uNK) cells and macrophages, have been found in the wall of human spiral arteries during pregnancy. They produce growth factors, proteases, and many other factors involved in regulating trophoblast invasion and spiral artery remodeling [87,88]. The uNK cells function is directly regulated by estrogens that bind to ER46, the predominant ER isoform, localized in the uNK cells membrane [89].

**Figure 1:**
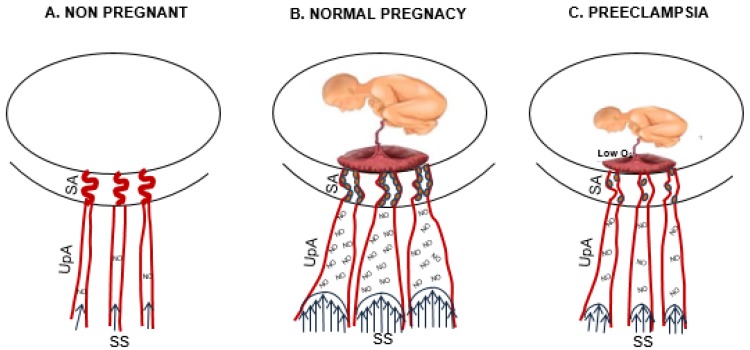
Uterine artery changes from the nonpregnant to pregnant state and in preeclampsia. The drawing shows the changes of uterine arteries from nonpregnancy (**A**) to pregnancy (**B**) and in preeclampsia (**C**). In normal pregnancy, the deep cytotrophoblast (full small circle) invasion of spiral arteries (SA) dilates SA and increases shear stress (SS) in upstream arteries (UpA), which increases nitric oxide (NO) production and vasodilation. (**C**) Placental hypoxia (low O_2_) in preeclampsia abrogates pregnancy-adaptation (spiral arteries invasion lead to shear stress lead to NO production leads to vasodilation) with consequent inhibition of the fetoplacental unit growth.

Spiral artery changes decrease distal resistance and increase blood flow into the placenta, thereby accelerating blood flow in upstream arteries. The augmented blood velocity increases wall shear stress on the uterine endothelium and triggers a series of events that lead to outward (expansive) remodeling (Figure 1). This effect of shear stress has been well documented in other vascular beds (carotids [90]; mesenteric circulation [91]; aorta [92]) and is thought to involve nitric oxide release, which stimulates vasodilation and vascular growth.

### 5.2. Estrogenic Stimulation of VEGF Signaling

**Figure 2:**
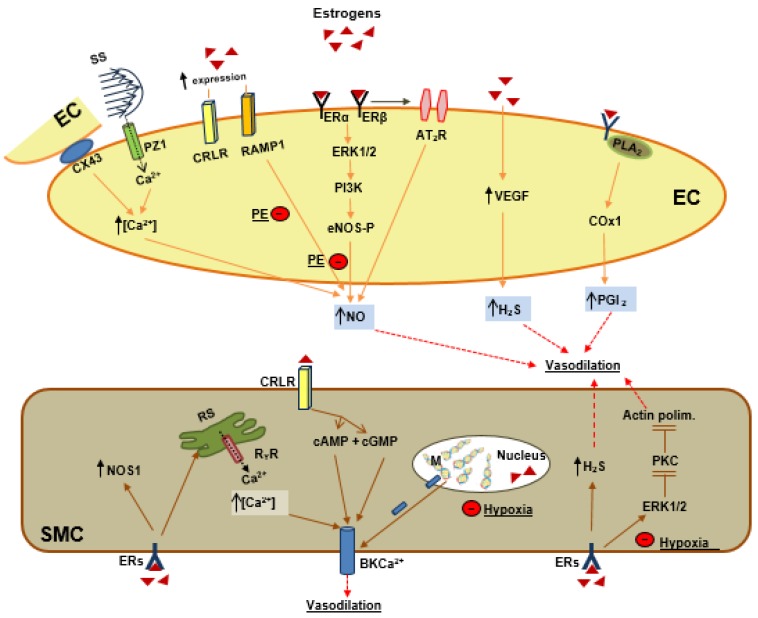
Estrogens act on endothelial and smooth muscle cells of uterine arteries in the pregnant state. In pregnancy, the high plasma levels of estrogens (full small triangles) activate several molecular pathways in uterine artery endothelial cells (EC) and in smooth muscle cells (SMC).

In the uterine vasculature, VEGF is the major growth factor mediator of estrogen’s effects. The VEGF family includes several isoforms (VEGF120, VEGF164, VEGF188, and VEGF205), with VEGF120 and VEGF164 being the main ones detected in the *rat* uterine vasculature [93]. Experiments in *monkey*, in vitro and in vivo, have shown that the increase in circulating estrogens during pregnancy elevates the expression of VEGFs and their receptors [94]. In UA of pregnant *rats* VEGF expression was 80% above that of nonpregnant animals. [93]. Also, in *mares*, quantitative RT-PCR revealed a greater abundance of VEGF mRNA during pregnancy [95]. VEGF contributes to uterine vascular remodeling by regulating different processes such as endothelial proliferation, vasodilation, and permeability [77,93,96]. In *humans*, estrogens induce proliferation of endometrial EC by VEGF and this effect was higher than in other tissues. The higher growth response and enhanced responsiveness to VEGF is likely due to either an increased affinity or elevated number of VEGF receptors [77]. In ovariectomized *mice*, 17β-estradiol (E2) increased the effects of VEGF in uterine arteries by a paracrine mechanism that resulted in increased expression of its receptor FLK-1/KDR (VEGFR2), a tyrosine kinase [97]. VEGF-induced vasodilation of UA was significantly higher in vessels from pregnant vs. nonpregnant *rats*, and this effect was completely inhibited by endothelial removal and also by the tyrosine kinase inhibitor genistein [93]. VEGF-stimulated eNOS expression in UAEC from pregnant *ewes* to a greater extent than in those from nonpregnant *ewes* in a Ca^2+^-dependent manner [98]. Estrogen-induced augmentation of VEGF effects on UA likely accounts for the capacity of estrogens to induce vasodilation (Figure 2), promote EC proliferation, and increase UPBF [99]. In summary, considerable evidence suggests that the estrogen-VEGF-NO pathway plays an important role in uterine vasodilation, thereby decreasing uterine vascular resistance and inducing uterine hemodynamic changes associated with pregnancy.

### 5.3. Estrogen Effects on Matrix Metalloproteinases (MMPs)

Uterine vascular remodeling during pregnancy also involves structural reorganization, including changes in the extracellular matrix (ECM). It has been reported that in UA, collagen and elastin, the main components of the extracellular matrix, change in a way that increases distensibility in pregnancy [81,100]. The main regulators of the ECM are the matrix metalloproteinases (MMPs), a family of proteases that degrade the ECM and connective tissue proteins and play an important role in the tissue remodeling that accompanies the rapid growth, differentiation, and structural adaptations of UA. The MMPs family includes several members and, in UA, MMP-2, MMP-3, MMP-7, MMP-9, MMP-12, and MMP-13 are all increased by late pregnancy [101]. In pregnant *bitches*, a significant positive correlation has been demonstrated between the expression of MMPs and the plasma concentration of estrogen [102], suggesting possible regulation of MMPs by estrogens. In support of this, several studies have shown an upregulation of MMPs expression by estrogen [32]. NO and MMPs pathway activation could play a role in shear stress-induced vascular remodeling, as suggested in resistance arteries where high flow-induced diameter enlargement and wall hypertrophy were associated with the sequential activation of eNOS and MMP9 [103].

## 6. Estrogens and Vascular Tone Regulation

Estrogens play a pivotal role in the regulation of UA vascular tone, a key determinant of appropriate UPBF. In particular, estrogens reduce vascular tone and augment UPBF through a direct action on both EC and SMC.

### 6.1. Estrogen and Endothelial Signaling

The exact pathway by which shear stress is sensed and transduced by the UA endothelium is not known, particularly in terms of which molecular or cellular structures act as mechanosensors. Based on a study in *rats*, we recently reported that the Piezo 1 channel is expressed in UA endothelium, is upregulated during pregnancy, and its stimulation by shear stress results in vasodilation that involves increases in endothelial calcium and release of NO (Figure 2) [104]. In *ovine* UAEC it was shown that shear stress induces Ca^2+^-mediated NO production is connexin 43 dependent (Figure 2) [105]. Besides this indirect mechanism, estrogens can also induce a rapid synthesis of NO by the activation of plasma membrane-associated ERs. Studies in UA shown that estradiol-17β binds to ERs localized on the EC plasmalemma and rapidly (within 2 min) increases NO production secondary to ERK 1/2 and PI3K/PKB activation, leading to eNOS phosphorylation (Figure 2) [69,106,107,108]. eNOS phosphorylation occurs via ERα and ERβ, independently from each other. In particular, ERα activation induced an increase in (Ser1177) eNOS and (Ser635) eNOS and a decrease in (Thr495) eNOS. In contrast, ER-beta activation only reduced (Thr495) eNOS.

In addition to the mechanism above reported, the metabolite of Estrogen 17β, 2-hydroxyestradiol (2-OHE_2_), in *ovine* UAEC, increases NO production via adrenergic receptors [109]

NO is an endogenous, powerful vasodilator whose production in the UA increases significantly during pregnancy and is considered to be the major actor in mediating gestational uterine vascular remodeling. We have shown in pregnant *rats* that treatment with L-NAME, a specific inhibitor of NO, prevented uterine vascular circumferential growth [5]. In addition, a study in pregnant eNOS-knockout *mice* showed that uterine radius, cross sectional area, and SMC proliferation were all significantly smaller vs. wild type controls [82].

Taken together, the above results suggest a pivotal role for NO in maternal gestational uterine vascular (outward) remodeling, and its abrogation may explain the impaired pregnancy outcome observed in both *rats* and *mice* [5,82].

In addition to NO production, estrogens also induce a rapid synthesis of PGI2 as demonstrated in *ovine* uterine EC where estradiol-17β acts via primarily ER-α, while its metabolites act via ERs-independent mechanisms that enhance PGI2 production by upregulating phospholipase A (2) and cyclooxygenase-1 expression (Figure 2) [58].

### 6.2. Estrogens as Endogenous Vasodilator Peptides

Pregnancy-reduced uterine vascular tone is also accomplished by the vasodilator effect of angiotensin II via angiotensin type-2 receptor (AT_2_R) whose expression is upregulated in rat UA by pregnancy via ERβ in an endothelium-dependent manner (Figure 2). Consistently, estradiol stimulates AT_2_R expression in primary *human* UAEC from pregnant, but not nonpregnant women [110]. Estrogens also stimulate the production of the potent endogenous vasodilator, hydrogen sulfide (H_2_S), which is higher in EC and SMC of UA from pregnant women and during the estrus stage of the menstrual cycle [111]. In *human* UAEC, it was shown that VEGF upregulated H_2_S-forming enzymes, probably via the actions of VEGF (Figure 2) [112].

Further, estradiol increases UA sensitivity to the endogenous vasoactive peptides adrenomedullin (AM) and the related protein calcitonin gene-related peptide (CGRP) by upregulating their receptors. Calcitonin receptor-like receptor (CRLR) and receptor activity-modifying protein 1(RAMP1) [113] are expressed in EC and SMC. AM in the endothelium activates the NO-cGMP-K_Ca_ pathway and induces vasodilation (Figure 2) [114], while, CGRP vasodilation is NO-independent, although it is mediated by cyclic nucleotides (cGMP and cAMP) and Kca channels (Figure 2) [113]. AM vasodilation effect in chorionic arteries from preeclamptic women was significantly attenuated compared to normal pregnancy [115].

### 6.3. Estrogens and Smooth Muscle Cells Signaling

UASMC are a target for estrogens that exert direct, acute vasorelaxant effects mediated by different mechanisms. In non-pregnant rats, this is due to nitric oxide produced from muscle (NOS1) in an ER-independent manner (Figure 2) [116].

Along with raising the levels of NO, estrogens upregulate large-conductance Ca^2+^-activated K^+^ (BK_Ca_) channels [117,118]. In detail, estrogens upregulate Ca^2+^ release events through ryanodine receptor (RyR) channels in the sarcoplasmic reticulum of SMC. This type of Ca^2+^ release, termed sparks, activates BK_Ca_ channels, causing spontaneous transient outward currents (STOCs), and hyperpolarizing currents (Figure 2) [119]. Estrogenic modulation of BK_Ca_ activity is mediated by DNA demethylation, which upregulates the BKβ1 subunit and increases BK_Ca_ channels activity [120].

Further, another mechanism for estrogens inducing SMC vasodilation was shown in *ovine* UA, where 17β estradiol upregulated ERK1/2 expression, downregulated the PKC signaling pathway, reduced actin polymerization, and abrogated uterine vascular tone (Figure 2) [79,80].

In EC (from left to right in Figure 2): (1) Estrogens indirectly increase shear stress (SS) which activates piezo 1 channels (PZ1) and connexin 43 protein (CX43) and increases intracellular calcium (Ca^++^) and NO production. (2) Estrogens upregulate the receptors for adrenomedullin, RAMP1, and receptors for calcitonin gene-related peptide, CRLR, the former contributing to NO production. Estrogen by its receptors, ERα and ERβ, activates the ERK1/2-PI3K -eNOS phosphorylation (eNOS-P) pathway and increases NO production. (3) Estrogens by ERβ activate angiotensin II receptors (AT_2_R), which contributes to NO production; (4) Estrogens by upregulation of vascular endothelial growth factor (VEGF) induce hydrogen sulfide (H_2_S) production; (5) Estrogens activate the phospholipase A_2_ (PLA_2_)–cyclooxygenase 1 pathway to produce prostacyclin (PGI2). Preeclampsia (PE) inhibits the estrogens, RAMP1-NO and also ERs-ERK1/2-PI3K and eNOS-P-NO.

In SMC (from left to right in Figure 2), estrogens activate (a) nitric oxide synthase neuronal (NOS1); (b) calcium release from the sarcoplasmic reticulum (RS), which activates the big potassium calcium channel (BK_Ca++_); (c) CRLR, which by the cyclic nucleotides cAMP and cGMP activates BK_Ca++_; (d) demethylation of the BKβ1 subunit and activation of BK_Ca++;_ (e) H_2_S production; (f) ERK1/2, which inhibits PKC and actin polymerization. Hypoxia inhibits BKβ1 demethylation and also estrogens activation of ERK1/2.

## 7. Preeclampsia

### 7.1. Preeclampsia is Associated with Lower Estrogen

Several recent studies have reported lower plasma estrogen concentrations in preeclamptic women compared with those having a normal pregnancy [58,121,122]. Lower estrogens can be due to abnormal activity of the enzymes involved in their biosynthesis [123,124] as occurred in pregnant women with gestational diabetes and in obese women, both with high probability of developing PE [125,126]. In obese women with PE, the elevated plasma levels of leptin decreased placental steroid biosynthesis [127], consistent with an in vitro study where incubation of placental explants with leptin decreased steroid biosynthesis and estrogen production [128]. Also, the placental hypoxia of preeclamptic women reduces the expression of placental aromatase, a key enzyme in estrogens synthesis [129,130]. Hypoxia activates transcription factors that inhibit aromatase expression with aberrant synthesis of estrogen leading to uterine vascular dysfunction in PE [131,132]. One study showed that chronic inhibition of aromatase in the second half of *baboon* pregnancy significantly decreased maternal serum estrogens, although UPBF and fetal growth were maintained at normal levels [133].

The lower circulating estrogen in PE prompted researchers to validate estrogen levels as a biomarker but also as a potential treatment for PE. Results from studies that have considered estrogen concentrations in pregnancy with other high risk factors to predict the risk for development of PE showed only a modest sensitivity and specificity [134]. A therapeutic approach showed that short-term estradiol therapy ameliorates PE [135,136].

Low plasma estrogen concentrations were associated with shallower spiral artery invasion [137,138], insufficient placental development, and poor maternal–fetal exchange, (Figure 1). This situation is worsened by a reduction in ERs expression in target tissues such as cytotrophoblast and UAcells. Abnormal placentae show a lack of ERα expression in cytotrophoblast that inhibit the normal action of estrogen in the regulation of their differentiation and function [137]. Also, placental hypoperfusion decreased ERα expression in *ovine* UA [139], although contradictory results have been reported for ERα polymorphisms in PE [140,141]. In addition, insufficient UPBF and, therefore, placental ischemia induced an excess release of the antiangiogenic factor soluble fms-like tyrosine Kinase 1 (sFlt1) from cytotrophoblast, which possess a unique ability to enhance sFlt-1 production in a low oxygen environment [142]. sFlt1 is the soluble form of VEGFR1 (Flt-1) that together with VEGR2 are functional receptors for placental growth factor (PlGF) and VEGF. In many preeclamptic patients, as well as in animal models of PE, PlGF–VEGFR1 and VEGF–VEGFR2 interactions are disturbed by excess amounts of sFlt1, a natural PlGF/VEGF antagonist [143,144,145]. In women with preeclampsia, the higher levels of sFlt-1 levels are associated with higher uterine artery pulsatility indices (PI, an index of uterine vascular resistance) and compromised uteroplacental hemodynamics [146,147]. sFlt1 can compromise UPBF by MMPs pathways, as shown in an animal model of PE where sFlt1 administration decreased MMP2 and MMP9 expression and increased arterial collagen content and vascular stiffness, [148,149]. The sFlt1 effect was reversed by administration of VEGF or PlGF, suggesting that growth factor–MMP pathways are involved in the regulation of vascular biomechanical properties [149,150]. Further, the impaired cytotrophoblast invasion of spiral arteries secondary to the low concentration of estrogen in PE can decrease uterine shear stress, although, to our knowledge, a direct correlation between the extent of spiral artery invasion and shear stress intensity has not been established. In UA from preeclamptic women, shear stress-mediated release of nitric oxide is impaired [151]. Because NO plays a key role in gestational uterine vascular remodeling, reduced NO levels may well explain the diminished remodeling reported in PE (Figure 1). A likely mechanism involves altered MMP/TIMP activity, as we have shown in pregnant rats that the inhibition of NO inhibited MMP2, reduced expansive remodeling, and significantly increased arterial wall collagen and elastin content relative to normal pregnancy [100].

### 7.2. Preeclampsia and Vascular Tone

In PE UA, vascular tone is higher compared to normal pregnancy due to decreased endothelium-dependent relaxation, partly as a consequence of lowered response to NO-mediated dilation and augmented response to numerous constrictors [152,153]. Several mechanisms have been reported in PE that can explain the reduced vascular tone. PE attenuates estrogens-mediated ERK1/2 signaling and results in upregulation of protein kinase C (PKC)-mediated actin polymerization (Figure 2) [154]. In addition, PKC mediates inhibition of BK_Ca_ channels and reduces relaxations of UA [155]. BK_Ca_ channel inhibition in PE also occurs by DNA methylation of the BKβ1 subunit, an epigenetic mechanisms that is promoted by hypoxia (Figure 2) [156,157].

## 8. Conclusions

In normal pregnancy, estrogens may, at least in part, drive maternal uterine vascular remodeling by influencing mechanisms associated with UA circumferential growth during pregnancy. In particular, estrogens play a role in mediating cytotrophoblast invasion of spiral arteries and subsequent increases in blood flow and shear stress. Estrogens have also been shown to act directly on EC to activate synthesis and production of vasodilators and growth factors such as NO and VEGF. The lower plasma estrogen levels and downregulation of ERs that have often been noted in preeclampsia may further attenuate spiral artery invasion, uterine vasodilation, and remodeling and contribute to placental underperfusion. A better understanding of the mechanisms by which estrogen regulates uterine vascular remodeling and uteroplacental hemodynamics could help in the development of new therapeutic approaches for the prevention and/or management of preeclampsia.

## References

[1] Palmer S.K., Zamudio S., Coffin C., Parker S., Stamm E., Moore L.G. (1992). Quantitative estimation of human uterine artery blood flow and pelvic blood flow redistribution in pregnancy. Obstet. Gynecol..

[2] Gerretsen G., Huisjes H.J., Elema J.D. (1981). The role of the spiral artieries in the placental bed in relation to preeclampsia and fetal growth retardation. Br. J. Obstet. Gynaecol..

[3] Van Beek E., Peeters L.L.H. (1998). Pathogenesis of pre-eclampsia: A comprehensive model. Obstet. Gynecol. Surv..

[4] Lydakis C., Beevers M., Beevers D.G., Lip G.Y. (2001). The prevalence of pre-eclampsia and obstetric outcome in pregnancies of normotensive and hypertensive women attending a hospital specialist clinic. Int. J. Clin. Pract..

[5] Osol G., Mandalà M. (2009). Maternal Uterine Vascular Remodeling During Pregnancy. Physiology.

[6] Ueland K. (1976). Maternal cardiovascular dynamics. VII. Intrapartum blood volume changes. Am. J. Obstet. Gynecol..

[7] Rockwell L.C., Vargas E., Moore L.G. (2003). Human physiological adaptation to pregnancy: Inter- and intraspecific perspectives. Am. J. Hum. Biol..

[8] Metcalfe J., Ueland K. (1974). Maternal cardiovascular adjustments to preg- nancy. Prog. Cardiovasc. Dis..

[9] Genbacev O.D., Prakobphol A., Foulk R.A., Krtolica A.R., Ilic D., Singer M.S., Yang Z.Q., Kiessling L.L., Rosen S.D., Fisher S.J. (2003). Trophoblast L-selectin-mediated adhesion at the maternal-fetal interface. Science.

[10] Zhou Y., Fisher S.J., Janatpour M., Genbacev O., Dejana E., Wheelock M., Damsky C.H. (1997). Human cytotrophoblasts adopt a vascular phenotype as they differentiate. A strategy for successful endovascular invasion?. J. Clin. Invest..

[11] Thaler I., Manor D., Itskovitz J., Rottem S., Levit N., Timor-Tritsch I., Brandes J.M. (1990). Changes in uterine blood flow during human pregnancy. Am. J. Obstet. Gynecol..

[12] Bjellin L., Sjoquist P.O., Carter A.M. (1975). Uterine, maternal placental and ovarian blood flow throughout pregnancy in the guinea pig. Zeitschrift fur Geburtshilfe und Perinatologie.

[13] Dowell R.T., Kauer C.D. (1997). Maternal hemodynamics and uteroplacental blood flow throughout gesta- tion in conscious rats. Meth. Find. Exp. Clin. Pharmacol..

[14] Lees M.H., Hill J.D., Ochsner A.J., Thomas C.L., Novy M.J. (1971). Maternal placental and myometrial blood flow of the rhesus monkey during uterine contractions. Am. J. Obstet. Gynecol..

[15] Rosenfeld C.R., Morriss F.H., Makowski E.L., Meschia G., Battaglia F.C. (1974). Circulatory changes in the reproductive tissues of ewes during pregnancy. Gynecol. Invest..

[16] Lunell N.O., Nylund L.E., Lewander R., Sarby B. (1982). Uteroplacental blood flow in pre-eclampsia measurements with indium-113m and a computer-linked camera. Clin. Exp. Hypertens. B.

[17] Zamudio S., Palmer S.K., Droma T., Stamm E., Coffin C., Moore L.G. (1995). Effect of altitude on uterine artery blood flow during normal pregnancy. J. Appl. Physiol..

[18] Chang K., Xiao D., Huang X., Xue Z., Yang S., Longo L.D., Zhang L. (2010). Chronic hypoxia inhibits sex steroid hormone-mediated attenuation of ovine uterine arterial myogenic tone in pregnancy. Hypertension.

[19] Charles S.M., Julian C.G., Vargas E., Moore L.G. (2014). Higher **estrogen** levels during **pregnancy** in Andean than European residents of high altitude suggest differences in aromatase activity. J. Clin. Endocrinol. Metab..

[20] Julian C.G., Wilson M.J., Lopez M., Yamashiro H., Tellez W., Rodriguez A., Bigham A.W., Shriver M.D., Rodriguez C., Vargas E. (2009). Augmented uterine artery blood flow and oxygen delivery protect Andeans from altitude-associated reductions in fetal growth. Am. J. Physiol. Regul. Integr. Comp. Physiol..

[21] Mucci L.A., Lagiou P., Tamimi R.M., Hsieh C.C., Adami H.O., Trichopoulos D. (2003). Pregnancy estriol, estradiol, progesterone and prolactin in relation to birth weight and other birth size variables (United States). Cancer Causes Control.

[22] Bernstein I.M.L., Ziegler W.F., Leavitt T., Badger G.J. (2002). Uterine artery hemodynamic adaptations through the menstrual cycle into early pregnancy. Obstet. Gynecol..

[23] Magness R.R., Phernetton T.M., Gibson T.C., Chen D.B. (2005). Uterine blood flow responses to ICI 182 780 in ovariectomized oestradiol-17beta-treated, intact follicular and pregnant sheep. J. Physiol..

[24] Magness R.R., Rosenfeld C.R. (1989). Local and systemic estradiol-17 beta: Effects on uterine and systemic vasodilation. Am. J. Physiol. Endocrinol. Metab..

[25] Mandalà M., Osol G. (2012). Physiological remodelling of the maternal uterine circulation during pregnancy. Basic Clin. Pharmacol. Toxicol..

[26] Mabie W.C., DiSessa T.G., Crocker L.G., Sibai B., Arheart K.L. (1994). A longitudinal study of cardiac output in normal human pregnancy. Am. J. Obstet. Gynecol..

[27] Mone S.M., Sanders S., Colan S.D. (1996). Control mechanisms for physiological hypertrophy of pregnancy. Circulation.

[28] Caulin-Glaser T., Garcıa-Cardena G., Sarrel P., Sessa W.C., Bender J.R. (1997). 17 beta-estradiol regulation of human endothelial cell basal nitric oxide release, independent of cytosolic Ca2+ mobilization. Circ. Res..

[29] Storment J.M., Meyer M., Osol G. (2000). Estrogen augments the vaso- dilatory effects of vascular endothelial growth factor in the uterine circulation of the rat. Am. J. Obstet. Gynecol..

[30] Stice S.L., Ford S.P., Rosazza J.P., Van Orden D.E. (1987). Interaction of 4- hydroxylated estradiol and potential-sensitive Ca2+ channels in altering uterine blood flow during the estrous cycle and early pregnancy in gilts. Biol. Reprod..

[31] Salom J.B., Burguete M.C., Pèrez-Asensio F.J., Torregrosa G., Alborch E. (2001). Relaxant effects of 17-beta-estradiol in cerebral arteries through Ca(2+) entry inhibition. J. Cereb. Blood Flow Metab..

[32] Dang Y., Li W., Tran V., Khalil R.A. (2013). EMMPRIN-mediated induction of uterine and vascular matrix metalloproteinases during pregnancy and in response to estrogen and progesterone. Biochem. Pharmacol..

[33] Shifren J.L., Tseng J.F., Zaloudek C.J., Ryan I.P., Meng Y.G., Ferrara N., Jaffe R.B., Taylor R.N. (1996). Ovarian steroid regulation of vascular endothelial growth factor in the human endometrium: Implications for angiogenesis during the menstrual cycle and in the patho- genesis of endometriosis. J. Clin. Endocrinol. Metab..

[34] Mueller M.D., Vigne J.L., Minchenko A., Lebovic D.I., Leitman D.C., Taylor R.N. (2000). Regulation of vascular endothelial growth factor (VEGF) gene transcription by estrogen receptors alpha and beta. Proc. Natl. Acad. Sci. USA..

[35] Menendez D., Inga A., Snipe J., Krysiak O., Schonfelder G., Resnick M.A. (2007). A single-nucleotide polymorphism in a half-binding site creates p53 and estrogen receptor control of vascular endothelial growth factor receptor 1. Mol. Cell Biol..

[36] Gargett C.E., Zaitseva M., Bucak K., Chu S., Fuller P.J., Rogers P.A.W. (2002). 17Beta-estradiol up-regulates vascular endothelial growth factor receptor-2 expression in human myometrial microvascular en- dothelial cells: Role of estrogen receptor-alpha and –beta. J. Clin. Endocrinol. Metab..

[37] Holmes D.I.R., Zachary I. (2004). Placental growth factor induces FosB and c-Fos gene expression via Flt-1 receptors. FEBS Lett..

[38] Rauschemberger M.B., Sandoval M.J., Massheimer V.L. (2011). Cellular and molecular actions displayed by estrone on vascular endothelium. Mol. Cell Endocrinol..

[39] Torgersen K.L., Curran C.A. (2006). A systematic approach to the physiologic adaptations of pregnancy. Crit. Care Nurs..

[40] Chang J., Streitman D. (2012). Physiologic adaptations to pregnancy. Neurol. Clin..

[41] Longo L.D. (1983). Maternal blood volume and cardiac output during pregnancy: A hypothesis of endocrinologic control. Am. J. Physiol..

[42] Cipolla M., Osol G. (1994). Hypertrophic and hyperplastic effects of pregnancy on the rat uterine arterial wall. Am. J. Obstet. Gynecol..

[43] Pijnenborg R., Robertson W.B., Brosens I., Dixon G. (1981). Review article: Trophoblast invasion and the establishment of haemochorial placentation in man and laboratory animals. Placenta.

[44] Wallace A.E., Fraser R., Cartwright J.E. (2012). Extravillous trophoblast and decidual natural killer cells: A remodelling partnership. Hum. Reprod. Update.

[45] Whitley G.S.J., Cartwright J.E. (2009). Trophoblast-mediated spiral artery remodelling: A role for apoptosis. J. Anat..

[46] Kliman H.J. (1999). Trophoblast to Human Placenta.

[47] Moll W. (2003). Structure adaptation and blood flow control in the uterine arterial system after hemochorial placentation. Eur. J. Obstet. Gynecol. Reprod. Biol..

[48] Page K.L., Celia G., Leddy G., Taatjes D.J., Osol G. (2002). Structural remodeling of rat uterine veins in preg- nancy. Am. J. Obstet. Gynecol..

[49] Gokina N.I., Mandalà M., Osol G. (2003). Induction of localized differences in rat uterine radial artery behav- ior and structure during gestation. Am. J. Obstet. Gynecol..

[50] Osol G., Cipolla M. (1993). Pregnancy-induced changes in the three-dimensional mechanical properties of pressurized rat uteroplacental (radial) arteries. Am. J. Obstet. Gynecol..

[51] Poston L. (1997). The control of blood flow to the placenta. Exp. Physiol..

[52] Griendling K.K., Fuller E.O., Cox R.H. (1985). Pregnancy- induced changes in sheep uterine and carotid arteries. Am. J. Physiol. Heart Circ. Physiol..

[53] MacKenzie S.M., Huda S.S., Sattar N., Fraser R., Connell J.M.C., Davies E. (2008). Depot-specific steroidogenic gene transcription in hu- man adipose tissue. Clin. Endocrinol. (Oxf)..

[54] Devroey P., Camus M., Palermo G., Smitz J., Van Waesberghe L., Wisanto A., Wijbo I., Van Steirteghem A.C. (1990). Placental production of estradiol and progesterone after oocyte donation in patients with primary ovarian failure. Am. J. Obstet. Gynecol..

[55] Oakey R.E. (1970). The progressive increase in estrogen production in human pregnancy: An appraisal of the factors responsible. Vitam. Horm..

[56] Bausero P., Ben-Mahdi M., Mazucatelli J., Bloy C., Perrot-Applanat M. (2000). Vascular endothelial growth factor is modulated in vascular muscle cells by estradiol, tamoxifen, and hypoxia. Am. J. Physiol. Heart Circ. Physiol..

[57] Calzada-Mendoza C.C., Sanchez E.C., Campos R.R., Becerril A.M., Madrigal E.B., Sierra A.R., Mendez E.B., Ocharan E.H., Herrera N.G., Ceballos-Reyes G. (2006). Differential aromatase (CYP19) expression in human arteries from normal and neoplasic uterus: An immunohistochemical and in situ hybridization study. Front. Biosci..

[58] Jobe S.O., Ramadoss J., Wargin A.J., Magness R.R. (2013). Estradiol-17b and its cytochrome P450- and catechol-O-methyltransferase-derived metabolites selectively stimulate production of prostacyclin in uterine artery endothelial cells: Role of estrogen receptor-a versus estrogen receptor-b. Hypertension.

[59] Peter M., Dorr H.G., Sippell W.G. (1994). Changes in the concentrations of dehydroepiandrosterone sulfate and estriol in maternal plasma during pregnancy: A longitudinal study in healthy women throughout gestation and at term. Horm. Res..

[60] Rivarola M.A., Forest M., Migeon C.J. (1968). Testosterone, androstenedione and dehydroepiandrosterone in plasma during pregnancy and at delivery: Concentration and protein binding. J. Clin. Endocrinol. Metab..

[61] Dubey R.K., Tofovic S.P., Jackson E.K. (2004). Cardiovascular pharmacology of estradiol metabolites. J. Pharmacol. Exp. Ther..

[62] Mullis P.E., Yoshimura N., Kuhlmann B., Lippuner K., Jaeger P., Harada H. (1997). Aromatase deficiency in a female who is compound heterozygote for two new point mutations in the P450arom gene: Impact of estrogens on hypergonadotropic hypogonadism, multicystic ovaries, and bone densitometry in childhood. J. Clin. Endocrinol. Metab..

[63] Ludwikowski B., Heger S., Datz N., Richter-Unruh A., Gonzàlez R. (2013). Aromatase deficiency: Rare cause of virilization. Eur. J. Pediatr. Surg..

[64] Levitz M., Young B.K. (1977). Estrogens in pregnancy. Vitam. Horm..

[65] Abbassi-Ghanavati M., Greer L.G., Cunningham F.G. (2009). Pregnancy and laboratory studies: A reference table for clinicians. Obstet. Gynecol..

[66] Kamat A., Alcorn J.L., Kunczt C., Mendelson C.R. (1998). Characterization of the regulatory regions of the human aromatase (P450arom) gene involved in placenta-specific expression. Mol. Endocrinol..

[67] Schultz J.R., Petz L.N., Nardulli A.M. (2005). Cell- and ligand-specific regulation of promoters containing activator protein-1 and Sp1 sites by estrogen receptors alpha and beta. J. Biol. Chem..

[68] Heldring N., Isaacs G.D., Diehl A.G., Sun M., Cheung E., Ranish J.A., Kraus W.L. (2011). Multiple sequence-specific DNA-binding proteins mediate estrogen receptor signaling through a tethering pathway. Mol. Endocrinol..

[69] Simoncini T., Genazzani A.R., Liao J.K. (2002). Non genomic mechanisms of endothelial nitric oxide synthase activation by the selective estrogen receptor modulator raloxifene. Circulation.

[70] Tessier C., Deb S., Prigent-Tessier A., Ferguson-Gottschall S., Gibori G.B., Shiu R.P., Gibori G. (2000). Estrogen receptors alpha and beta in rat decidua cells: Cell-specific expression and differential regulation by steroid hormones and prolactin. Endocrinology.

[71] Bukovsky A., Caudle M.R., Cekanova M., Fernando R.I., Wimalasena J., Foster J.S., Henley D.C., Elder R.F. (2003). Placental expression of estrogen receptor beta and its hormone binding variant–comparison with estrogen receptor alpha and a role for estrogen receptors in asymmetric division and differentiation of estrogen-dependent cells. Reprod. Biol. Endocrinol..

[72] Perrot-Applanat M., Deng M., Fernandez H., Lelaidier C., Meduri G., Bouchard P. (1994). Immunohistochemical localization of estradiol and progesterone receptors in human uterus throughout preg- nancy: Expression in endometrial blood vessels. J. Clin. Endocrinol. Metab..

[73] Byers M.J., Zangl A., Phernetton T.M., Lopez G., Chen D.B., Magness R.R. (2005). Endothelial vasodilator production by ovine uterine and systemic arteries: Ovarian steroid and pregnancy control of Eralpha and Erbeta levels. J. Physiol..

[74] Barnea E.R., MacLusky N.J., Naftolin F. (1983). Kinetics of catechol estrogen-estrogen receptor dissociation: A possible factor un- derlying differences in catechol estrogen biological activity. Steroids.

[75] Liao W.X., Magness R.R., Chen D.B. (2005). Expression of estrogen receptors-alpha and -beta in the pregnant ovine uterine artery endothelial cells in vivo and in vitro. Biol. Reprod..

[76] Tropea T., De Francesco E.M., Rigiracciolo D., Maggiolini M., Wareing M., Osol G., Mandalà M. (2015). Pregnancy Augments G Protein Estrogen Receptor (GPER) Induced Vasodilation in Rat Uterine Arteries via the Nitric Oxide - cGMP Signaling Pathway. PLoS ONE.

[77] Iruela-Arispe M., Rodriguez-Manzaneque J.C., Abu-Jawdeh G. (1999). Endometrial endothelial cells express estrogen and progesterone receptors and exhibit a tissue specific response to angiogenic growth factors. Microcirculation.

[78] Pastore M.B., Jobe S.O., Ramadoss J., Magness R.R. (2012). Estrogen receptor-α and estrogen receptor-β in the uterine vascular endothelium during pregnancy: Functional implications for regulating uterine blood flow. Semin. Reprod. Med..

[79] Xiao D., Huang X., Yang S., Zhang L. (2009). Direct chronic effect of steroid hormones in attenuating uterine arterial myogenic tone: Role of protein kinase c/extracellular signal-regulated kinase 1/2. Hypertension.

[80] Xiao D., Huang X., Yang S., Longo L.D., Zhang L. (2010). Pregnancy downregulates actin polymerization and pressure-dependent myogenic tone in ovine uterine arteries. Hypertension.

[81] Lydrup M.L., Fernö M. (2003). Correlation between estrogen receptor alpha expression, collagen content and stiffness in human uterine arteries. Acta Obstet. Gynecol. Scand..

[82] van der Heijden O.W., Essers Y.P., Fazzi G., Peeters L.L., De Mey J.G., van Eys G.J. (2005). Uterine artery remodeling and reproductive performance are impaired in endothelial nitric oxide synthase-deficient mice. Biol. Reprod..

[83] Makinoda S., Moll W. (1986). Deoxyribonucleic acid synthesis in mesometrial arteries of guinea pigs during oestrous cycle, pregnancy and treatment with oestradiol benzoate. Placenta.

[84] Moll W., Nienartowicz A., Hees H., Wrobel K.H., Lenz A. (1988). Blood flow regulation in the uteroplacental arteries. Troph Res..

[85] Chang K., Lubo Z. (2008). Review article: Steroid hormones and uterine vascular adaptation to pregnancy. Reprod. Sci..

[86] Bonagura T.W., Pepe G.J., Enders A.C., Albrecht E.D. (2008). Suppression of Extravillous Trophoblast Vascular Endothelial Growth Factor Expression and Uterine Spiral Artery Invasion by Estrogen during Early Baboon Pregnancy. Endocrinology.

[87] Robson A., Harris L.K., Innes B.A., Lash G.E., Aljunaidy M.M., Aplin J.D., Baker P.N., Robson S.C., Bulmer J.N. (2012). Uterine natural killer cells initiate spiral artery remodeling in human pregnancy. FASEB J..

[88] Lash G.E., Pitman H., Morgan H.L., Innes B.A., Agwu C.N., Bulmer J.N. (2016). Decidual macrophages: Key regulators of vascular remodeling in human pregnancy. J. Leukoc. Biol..

[89] Gibson D.A., Esnal-Zufiaurre A., Bajo-Santos C., Collins F., Critchley H.O.D., Saunders P.T.K. (2020). Profiling the expression and function of oestrogen receptor isoform ER46 in human endometrial tissues and uterine natural killer cells. Hum. Reprod..

[90] Brownlee R.D., Langille B.L. (1991). Arterial adaptations to altered blood flow. Can. J. Physiol. Pharmacol..

[91] Tarhouni K., Guihot A.L., Freidja M.L., Toutain B., Henrion B., Baufreton C., Pinaud F., Procaccio V., Grimaud L., Ayer A. (2013). Key role of estrogens and endothelial estrogen receptor α in blood flow-mediated remodeling of resistance arteries. Arterioscler. Thromb. Vasc. Biol..

[92] Zarins C.K., Zatina M.A., Giddens D.P., Ku D.N., Glagov S. (1987). Shear stress regulation of artery lumen diameter in experimental atherogenesis. J. Vasc. Surg..

[93] Ni Y., May V., Brees K., Osol G. (1997). Pregnancy augments uteroplacental vascular endothelial growth factor gene expression and vasodilator effects. Am. J. Physiol..

[94] Greb R.R., Heikinheimo O., Williams R.F., Hodgen G.D., Goodman A.L. (1997). Vascular endothelial growth factor in primate endometrium is regulated by oestrogen-receptor and progesterone-receptor ligands in vivo. Hum. Reprod..

[95] Silva L.A., Klein C., Ealy A.D., Sharp D.C. (2011). Conceptus-mediated endometrial vascular changes during early pregnancy in mares: An anatomic, histomorphometric, and vascular endothelial growth factor receptor system immunolocalization and gene expression study. Reproduction.

[96] Rockwell L.C., Pillai S., Olson C.E., Koos R.D. (2002). Inhibition of vascular endothelial growth factor/vascular permeability factor action blocks estrogen-induced uterine edema and implantation in rodents. Biol. Reprod..

[97] Hervè M.A., Meduri G., Petit F.G., Domet T.S., Lazennec G., Mourah S., Perrot-Applanat M. (2006). Regulation of the vascular endothelial growth factor (VEGF) receptor Flk-1/KDR by estradiol through VEGF in uterus. J. Endocrinol..

[98] Boeldt D.S., Grummer M.A., Magness R.R., Bird I.M. (2014). Altered VEGF-stimulated Ca2+ signaling in part underlies pregnancy-adapted eNOS activity in UAEC. J. Endocrinol..

[99] Mehta V., Abi-Nader K.N., Shangaris P., Shaw S.W., Filippi E., Benjamin E., Boyd M., Peebles D.M., Martin J., Zachary I. (2014). Local over-expression of VEGF-DΔNΔC in the uterine arteries of pregnant sheep results in long-term changes in uterine artery contractility and angiogenesis. PLoS ONE.

[100] Hale S.A., Weger L., Mandalà M., Osol G. (2011). Reduced NO signaling during pregnancy attenuates outward uterine artery remodeling by altering MMP expression and collagen and elastin deposition. Am. J. Physiol. Heart Circ. Physiol..

[101] Kelly B.A., Bond B.C., Poston L. (2003). Gestational profile of matrix metalloproteinases in rat uterine artery. Mol. Hum. Reprod..

[102] Schafer-Somi S., Ali Aksoy O., Patzl M., Findik M., Erunal-Maral N., Beceriklisoy H.B., Polat B., Aslan S. (2005). The activity of matrix metalloproteinase-2 and -9 in serum of pregnant and non-pregnant bitches. Reprod. Domest. Anim..

[103] Dumont O., Loufrani L., Henrion D. (2007). Key role of the NO-pathway and matrix metalloprotease-9 in high blood flow-induced remodeling of rat resistance arteries. Arterioscler. Thromb. Vasc. Biol..

[104] John L., Ko N.L., Gokin A., Gokina N., Mandalà M., Osol G. (2018). The Piezo1 cation channel mediates uterine artery shear stress mechanotransduction and vasodilation during rat pregnancy. Am. J. Physiol. Heart Circ. Physiol..

[105] Morschauser T.J., Ramadoss J., Koch J.M., Yi F.X., Lopez G.E., Bird I.M., Magness R.R. (2014). Local effects of pregnancy on connexin proteins that mediate Ca2+-associated uterine endothelial NO synthesis. Hypertension.

[106] Chen D.B., Bird I.M., Zheng J., Magness R.R. (2004). Membrane estrogen receptor-dependent extracellular signal-regulated kinase pathway mediates acute activation of endothelial nitric oxide synthase by estrogen in uterine artery endothelial cells. Endocrinology.

[107] Pastore M.B., Talwar S., Conley M.R., Magness R.R. (2016). Identification of Differential ER-Alpha Versus ER-Beta Mediated Activation of eNOS in Ovine Uterine Artery Endothelial Cells. Biol. Reprod..

[108] Pang Y., Thomas P. (2017). Additive effects of low concentrations of estradiol-17β and progesterone on nitric oxide production by human vascular endothelial cells through shared signaling pathways. J. Steroid Biochem. Mol. Biol..

[109] Landeros R.V., Pastore M.B., Magness R.R. (2019). Effects of the Catechol and Methoxy Metabolites of 17β-Estradiol on Nitric Oxide Production by Ovine Uterine Artery Endothelial Cells. Reprod. Sci..

[110] Mishra J.S., Te Riele G.M., Qi Q.R., Lechuga T.J., Gopalakrishnan K., Chen D.B., Kumar S. (2019). Estrogen Receptor-β Mediates Estradiol-Induced Pregnancy-Specific Uterine Artery Endothelial Cell Angiotensin Type-2 Receptor Expression. Hypertension.

[111] Lechuga T.J., Qi Q.R., Magness R.R., Chen D.B. (2019). Ovine uterine artery hydrogen sulfide biosynthesis in vivo: Effects of ovarian cycle and pregnancy. Biol. Reprod..

[112] Zhang H.H., Chen J.C., Sheibani L., Lechuga T.J., Chen D.B. (2017). Pregnancy Augments VEGF-Stimulated In Vitro Angiogenesis and Vasodilator (NO and H2S) Production in Human Uterine Artery Endothelial Cells. J. Clin. Endocrinol. Metab..

[113] Gangula P.R., Thota C., Wimalawansa S.J., Bukoski R.D., Yallampalli C. (2003). Mechanisms involved in calcitonin gene-related Peptide-induced relaxation in pregnant rat uterine artery. Biol. Reprod..

[114] Ross G.R., Yallampalli U., Gangula P.R., Reed L., Sathishkumar K., Gao H., Chauhan M., Yallampalli C. (2010). Adrenomedullin relaxes rat uterine artery: Mechanisms and influence of pregnancy and estradiol. Endocrinology.

[115] Dong Y.L., Green K.E., Vegiragu S., Hankins G.D., Martin E., Chauhan M., Thota C., Yallampalli C. (2005). Evidence for decreased calcitonin gene-related peptide (CGRP) receptors and compromised responsiveness to CGRP of fetoplacental vessels in preeclamptic pregnancies. J. Clin. Endocrinol. Metab..

[116] Scott P.A., Tremblay A., Brochu M., St-Louis J. (2007). Vasorelaxant action of 17 -estradiol in rat uterine arteries: Role of nitric oxide synthases and estrogen receptors. Am. J. Physiol. Heart Circ. Physiol..

[117] Rosenfeld C.R., Roy T. (2012). Large conductance Ca2+-activated and voltage-activated K+ channels contribute to the rise and maintenance of estrogen-induced uterine vasodilation and maintenance of blood pressure. Endocrinology.

[118] Hu X.Q., Xiao D., Zhu R., Huang X., Yang S., Wilson S., Zhang L. (2011). Pregnancy upregulates large-conductance Ca(2+)-activated K(+) channel activity and attenuates myogenic tone in uterine arteries. Hypertension.

[119] Hu X.Q., Song R., Romero M., Dasgupta C., Huang X., Holguin M.A., Williams V., Xiao D., Wilson S.M., Zhang L. (2019). Pregnancy Increases Ca_2+_ Sparks/Spontaneous Transient Outward Currents and Reduces Uterine Arterial Myogenic Tone. Hypertension.

[120] Hu X.Q., Dasgupta C., Chen M., Xiao D., Huang X., Han L., Yang S., Xu Z., Zhang L. (2017). Pregnancy Reprograms Large-Conductance Ca2+-Activated K_+_ Channel in Uterine Arteries: Roles of Ten-Eleven Translocation Methylcytosine Dioxygenase 1-Mediated Active Demethylation. Hypertension.

[121] Pertegal M., Fenoy F.J., Hernàndez M., Mendiola J., Delgado J.L., Bonacasa B., Corno A., Lòpez B., Bosch V., Hernàndez I. (2016). Fetal Val108/158Met catechol-O-methyltransferase (COMT) polymorphism and placental COMT activity are associated with the development of preeclampsia. Fertil. Steril..

[122] Shen Z., Wu Y., Chen X., Chang X., Zhou Q., Zhou J., Ying H., Zheng J., Duan T., Wang K. (2014). Decreased maternal serum 2-methoxyestradiol levels are associated with the development of preeclampsia. Cell Physiol. Biochem..

[123] Hahnel M.E., Martin J.D., Michael C.A., Hahnel R. (1989). Metabolism of androstenedione by placental microsomes in pregnancy hypertension. Clin. Chim. Acta.

[124] Shimodaira M., Nakayama T., Sato I., Sato N., Izawa N., Mizutani Y., Furuya K., Yamamoto T. (2012). Estrogen synthesis genes CYP19A1, HSD3B1, and HSD3B2 in hypertensive disorders of pregnancy. Endocrine.

[125] Morisset A.S., Dubè M.C., Drolet R., Pelletier M., Labrie F., Luu-The V., Tremblay Y., Robitaille J., John Weisnagel S., Tchernof A. (2013). Androgens in the maternal and fetal circulation: Association with insulin resistance. J. Matern. Fetal Neonatal Med..

[126] Nestler J.E. (1987). Modulation of aromatase and P450 cholesterol sidechain cleavage enzyme activities of human placental cytotrophoblasts by insulin and insulin-like growth factor I. Endocrinology.

[127] Turgut A., Ozler A., Goruk N.Y., Tunç S.Y., Sak M.E., Evsen M.S., Evliyaoglu O., Gul T. (2015). Serum levels of the adipokines, free fatty acids, and oxidative stress markers in obese and non-obese preeclamptic patients. Clin. Exp. Obstet. Gynecol..

[128] Coya R., Martul P., Algorta J., Aniel-Quiroga M.A., Busturia M.A., Senaris R. (2006). Effect of leptin on the regulation of placental hormone secretion in cultured human placental cells. Gynecol. Endocrinol..

[129] Berkane N., Liere P., Lefevre G., Alfaidy N., Nahed R.A., Vincent J., Oudinet J.P., Pianos A., Cambourg A., Rozenberg P. (2018). Abnormal steroidogenesis and aromatase activity in preeclampsia. Placenta.

[130] Perez-Sepulveda A., Monteiro L.J., Dobierzewska A., España-Perrot P.P., Venegas-Araneda P., Guzmán-Rojas A.M., González M.I., Palominos-Rivera M., Irarrazabal C.E., Figueroa-Diesel H. (2015). Placental Aromatase Is Deficient in Placental Ischemia and Preeclampsia. PLoS ONE.

[131] Jiang B., Mendelson C.R. (2003). USF1 and USF2 mediate inhibition of human trophoblast differentiation and CYP19 gene expression by Mash-2 and hypoxia. Mol. Cell Biol..

[132] Hertig A., Liere P., Chabbert-Buffet N., Fort J., Pianos A., Eychenne B., Cambourg A., Schumacher M., Berkane N., Lefevre G. (2010). Steroid profiling in preeclamptic women: Evidence for aromatase deficiency. Am. J. Obstet. Gynecol..

[133] Aberdeen G.W., Baschat A.A., Harman C.R., Weiner C.P., Langenberg P.W., Pepe G.J., Albrecht E.D. (2010). Uterine and fetal blood flow indexes and fetal growth assessment after chronic estrogen suppression in the second half of baboon pregnancy. Am. J. Physiol. Heart Circ. Physiol..

[134] Stamilio D.M., Sehdev H.M., Morgan M.A., Propert K., Macones G.A. (2000). Can antenatal clinical and biochemical markers predict the development of severe preeclampsia?. Am. J. Obstet. Gynecol..

[135] Babic G.M., Markovic S.D., Varjacic M., Djordjevic N.Z., Nikolic T., Stojic I., Jakovljevic V. (2018). Estradiol decreases blood pressure in association with redox regulation in preeclampsia. Clin. Exp. Hypertens..

[136] Djordjević N.Z., Babić G.M., Marković S.D., Ognjanović B.I., Stajn A.S., Saicić Z.S. (2010). The antioxidative effect of estradiol therapy on erythrocytes in women with preeclampsia. Reprod. Toxicol..

[137] Bukovsky A., Cekanova M., Caudle M.R., Wimalasena J., Foster J.S., Henley D.C., Elder R.F. (2003). Expression and localization of estrogen receptor-alpha protein in normal and abnormal term placentae and stimulation of trophoblast differentiation by estradiol. Reprod. Biol. Endocrinol..

[138] Malassine A., Cronier L. (2002). Hormones and human trophoblast differentiation: A review. Endocrine.

[139] Dasgupta C., Chen M., Zhang H., Yang S., Zhang L. (2012). Chronic hypoxia during gestation causes epigenetic repression of the estrogen receptor-a gene in ovine uterine arteries via heightened promoter methylation. Hypertension.

[140] Molvarec A., Vèr A., Fekete A., Rosta K., Derzbach L., Derzsy Z., Karàdi I., Rigò J. (2007). Association between estrogen receptor alpha (ESR1) gene polymorphisms and severe preeclampsia. Hypertens. Res..

[141] Zhang J., Bai H., Liu X., Fan P., Liu R., Huang Y., Wang X., He G., Liu Y., Liu B. (2009). Genotype distribution of estrogen receptor alpha polymorphisms in pregnant women from healthy and preeclampsia populations and its relation to blood pressure levels. Clin. Chem. Lab. Med..

[142] Nagamatsu T., Fujii T., Kusumi M., Zou L., Yamashita T., Osuga Y., Momoeda M., Kozuma S., Taketani Y. (2004). Cytotrophoblasts Up-Regulate Soluble Fms-Like Tyrosine Kinase-1 Expression under Reduced Oxygen: An Implication for the Placental Vascular Development and the Pathophysiology of Preeclampsia. Endocrinology.

[143] Gilbert J.S., Babcock S.A., Granger J.P. (2007). Hypertension produced by reduced uterine perfusion in pregnant rats is associated with increased soluble fms-like tyrosine kinase-1 expression. Hypertension.

[144] Brockelsby J., Hayman R., Ahmed A., Warren A., Johnson I., Baker P. (1999). VEGF via VEGF receptor-1 (Flt-1) mimics preeclamptic plasma in inhibiting uterine blood vessel relaxation in pregnancy: Implications in the pathogenesis of preeclampsia. Lab. Invest..

[145] Khankin E.V., Mandalà M., Colton I., KarumanchI S.A., Osol G. (2012). Hemodynamic, vascular, and reproductive impact of FMS-like tyrosine kinase 1 (FLT1) blockade on the uteroplacental circulation during normal mouse pregnancy. Biol. Reprod..

[146] Savvidou M.D., Yu C.K., Harland L.C., Hingorani A.D., Nicolaides K.H. (2006). Maternal serum concentration of soluble fms-like tyrosine kinase 1 and vascular endothelial growth factor in women with abnormal uterine artery Doppler and in those with fetal growth restriction. Am. J. Obstet. Gynecol..

[147] Schlembach D., Wallner W., Sengenberger R., Stiegler E., Mörtl M., Beckmann M.W., Lang U. (2007). Angiogenic growth factor levels in maternal and fetal blood: Correlation with Doppler ultrasound parameters in pregnancies complicated by pre-eclampsia and intrauterine growth restriction. Ultrasound Obstet. Gynecol..

[148] Lin C., He H., Cui N., Ren Z., Zhu M., Khalil R.A. (2020). Decreased uterine vascularization and uterine arterial expansive remodeling with reduced matrix metalloproteinase-2 and -9 in hypertensive pregnancy. Am. J. Physiol. Heart Circ. Physiol..

[149] Li W., Mata K.M., Mazzuza M.Q., Khalil R.A. (2014). Altered matrix metalloproteinase-2 and -9 expression/activity links placental ischemia and anti-angiogenic sFlt-1 to uteroplacental and vascular remodeling and collagen deposition in hypertensive pregnancy. Biochem. Pharmacol..

[150] Ren Z., Cui N., Zhu M., Khalil R.A. (2018). Placental growth factor reverses decreased vascular and uteroplacental MMP-2 and MMP-9 and increased MMP-1 and MMP-7 and collagen types I and iV in hypertensive pregnancy. Am. J. Physiol. Heart Circ. Physiol..

[151] Kublickiene K.R., Lindblom B., Krüger K., Nisell H. (2000). Preeclampsia: Evidence for impaired shear stress-mediated nitric oxide release in uterine circulation. Am. J. Obstet. Gynecol..

[152] He M., Li F., Yang M., Fan Y., Beejadhursing R., Xie Y., Zhou Y., Deng D. (2018). Impairment of BKca channels in human placental chorionic plate arteries is potentially relevant to the development of preeclampsia. Hypertens. Res..

[153] Pascoal I.F., Lindheimer M.D., Nalbantian-Brandt C., Umans J.G. (1998). Preeclampsia selectively impairs endothelium-dependent relaxation and leads to oscillatory activity in small omental arteries. J. Clin. Invest..

[154] Xiao D., Huang X., Zhang L. (2012). Chronic hypoxia differentially up-regulates protein kinase C-mediated ovine uterine arterial contraction via actin polymerization signaling in pregnancy. Biol. Reprod..

[155] Xiao D., Zhu R., Zhang L. (2014). Gestational hypoxia up-regulates protein kinase C and inhibits calcium-activated potassium channels in ovine uterine arteries. Int. J. Med. Sci..

[156] Zhu R., Hu X.Q., Xiao D., Yang S., Wilson S.M., Longo L.D., Zhang L. (2013). Chronic Hypoxia Inhibits Pregnancy-Induced Upregulation of SK_Ca_ Channel Expression and Function in Uterine Arteries. Hypertension.

[157] Chen M., Xiao D., Hu X.Q., Dasgupta C., Yang S., Zhang L. (2015). Hypoxia Represses ER-α Expression and Inhibits Estrogen-Induced Regulation of Ca2+-Activated K+ Channel Activity and Myogenic Tone in Ovine Uterine Arteries: Causal Role of DNA Methylation. Hypertension.

